# EXTANT RESEARCH USING THE NATIONAL SURVEY OF OLDER AMERICANS ACT PARTICIPANTS: A SCOPING REVIEW

**DOI:** 10.1093/geroni/igae098.1039

**Published:** 2024-12-31

**Authors:** Tochukwu Okolie, Heather Menne

**Affiliations:** Miami University, Oxford, Ohio, United States; Miami University, Oxford, Ohio, United States

## Abstract

Since 2003, the National Survey of Older Americans Act Participants (NSOAAP) has been collected annually for programs funded by Title III of the Older Americans Act (OAA). Covering six program modules including case management, congregate meals, home-delivered meals, homemaker, transportation, and family caregiver support, the NSOAAP data provides rich information for uncovering important issues pertaining to older adults and caregivers who utilize these services. Regardless of its promising content for advancing aging studies in the United States, there is a dearth of scholarly literature that has utilized this data source. Hence, this scoping review sought to map out extant peer-reviewed empirical articles that used data from the NSOAAP. Eight databases were searched with no time restriction, resulting in 42 citations. Two duplicate citations were resolved, leaving 40 articles for the initial title and abstract review. Additional three citations were identified through online searches and reference list review. After the title and abstract review, 33 articles were excluded because they did not meet the inclusion criteria. This study and its findings are based on 10 peer-reviewed empirical studies. Findings from the review showed that while there is a dearth in overall empirical studies using the NSOAAP, case management, homemaker, and transportation were the least studied. Caregiver services and congregate meal services were analyzed in 4 studies and home-delivered meals in 3 studies. This scoping review highlights the need for more research to understand the impact of the OAA service as measured through the NSOAAP across time.

